# Challenges and Perspective in Integrated Multi-Omics in Gut Microbiota Studies

**DOI:** 10.3390/biom11020300

**Published:** 2021-02-17

**Authors:** Eric Banan-Mwine Daliri, Fred Kwame Ofosu, Ramachandran Chelliah, Byong H. Lee, Deog-Hwan Oh

**Affiliations:** 1 Department of Food Science and Biotechnology, Kangwon National University, Chuncheon 200-701, Korea; ericdaliri@kangwon.ac.kr (E.B.-M.D.); ofosufk17@kangwon.ac.kr (F.K.O.); ramachandran865@gmail.com (R.C.); 2 SportBiomics, Sacramento Inc., California, CA 95660, USA; byong.lee@mail.mcgill.ca

**Keywords:** Microbiome, biomarkers, multi-omics, metagenomics, metatranscriptomics

## Abstract

The advent of omic technology has made it possible to identify viable but unculturable micro-organisms in the gut. Therefore, application of multi-omic technologies in gut microbiome studies has become invaluable for unveiling a comprehensive interaction between these commensals in health and disease. Meanwhile, despite the successful identification of many microbial and host–microbial cometabolites that have been reported so far, it remains difficult to clearly identify the origin and function of some proteins and metabolites that are detected in gut samples. However, the application of single omic techniques for studying the gut microbiome comes with its own challenges which may be overcome if a number of different omics techniques are combined. In this review, we discuss our current knowledge about multi-omic techniques, their challenges and future perspective in this field of gut microbiome studies.

## 1. Introduction

**Figure 1:**
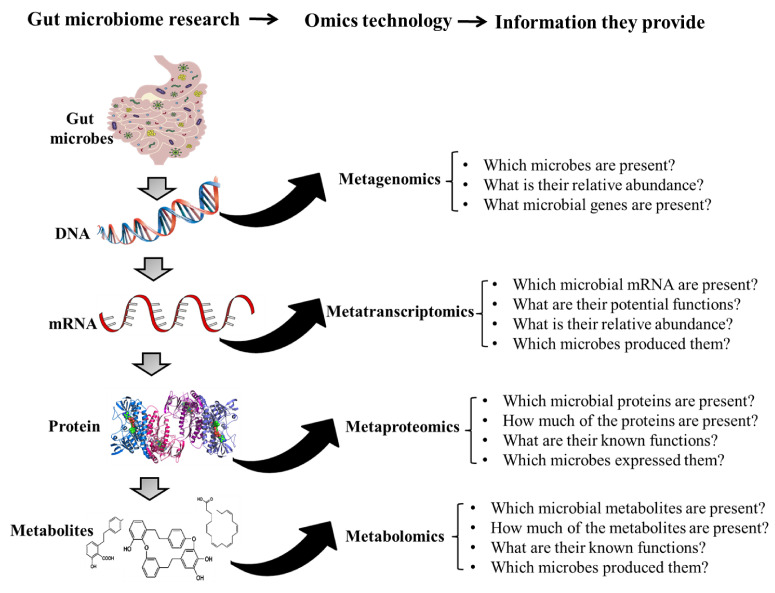
Omic technologies and the information they provide in gut-microbiome research.

The gut microbiota is very diverse and contains a large number of culturable and unculturable members which play critical roles in host health and disease. The members of the gut microbiota include archaea, bacteria, viruses, and fungi [1] and these organisms interact with each other and with the host. Metagenomic sequencing techniques have made it possible to study the microbial communities in the gut under different conditions and this helps to detect alterations that occur during disease conditions. This technique has been helpful in distinguishing healthy subjects from cancer [2], inflammatory bowel disease [3], as well as autism [4] patients. However, the presence of a microbe does not give any indication of its role in the gut. Also, the metabolic potentials of uncultured microbes are unknown and this makes metagenomics data alone inadequate in providing information about the gut microbial ecology [5]. Meanwhile, as only the DNA of live and active microbes are transcribed into RNA, analyzing gut microbial mRNA (metatranscriptomics) has become a robust technique for detecting and quantifying transcribed mRNA to predict their metabolic potentials [6]. Yet, since not all mRNAs are translated into proteins, metaproteomics, an analytical technique that can analyze gut microbial proteins in samples, is usually used to detect and quantify such proteins [7]. Other microbial metabolites such as lipids, carbohydrates, and some other biomolecules have also been shown to be essential for microbe–host interaction [8]. For this reason, some researchers apply metabolomics to identify gut microbial metabolites as well as host–microbe cometabolites which may help in unravelling the complex interaction between host and gut microbes [8]. The diagrammatic representation of several omic techniques and the information they provide are presented in Figure 1. The strengths and challenges in the application of different omic techniques are elaborated in the subsequent sections. 

## 2. Omic Techniques in Gut Microbiome Studies

### 2.1. Metagenomics

Metagenomics is a technique that sequences the genomes of microbes (archaea, bacteria, viruses, and fungi) present in a given sample [9]. Two approaches are commonly used, namely marker gene analysis and shotgun sequencing. In marker gene analysis, primers are designed to bind to highly conserved regions of a gene of interest (16S rRNA for bacteria and internal transcribed spacer for fungi) in order to identify the phylogenies of the microbial communities in a sample. Many studies have applied this approach in identifying gut bacteria [10] and fungi [11], and their relative proportions. However, since primers do not show equal affinities for all DNA sequences due to the variability in the primer-amplified regions, many biases are introduced during PCR amplification. More so, horizontal gene transfer can occur between microbes which may result in the transfer of informative sequences to unrelated microbes. This can lead to problems in correctly estimating the diversity of the microbial community. Marker gene analysis does not give any detailed information about the genes present in the microbes and so researchers who use this technique can only make associations between microbial populations and disease conditions [10,12,13]. Currently, many microbes present in the gut are unknown (i.e, their genomes have not been sequenced) and may therefore lack informative markers or primers [9,14,15] and yet contribute to health and disease. Such important microbes cannot be detected using marker gene sequencing. Despite the many limitations of marker gene analysis, many bioinformatics tools are available to predict the potential functions of gut microbes based on 16S rRNA data. Several platforms such including PICRUSt [16], Piphillin [17], PUMAA [18], iVikodak [19], and BURRITO [20] have been instrumental in inferring microbial functions based on 16S rRNA and this has promoted hypothesis testing. Shotgun sequencing however presents a detailed genetic and taxonomic information about the gut microbiota [21]. It is relatively more expensive and time consuming relative to marker gene analysis. The technique has been applied in analyzing uncultured bacteria [9], archaea [22], and viruses [23]. Shotgun sequencing profiles may present information about the potential function(s) of an entire microbial community at the gene level based on databases of already sequenced genomes, yet the information may not accurately represent the happenings in the gut at a given time. This is because all microbes whether metabolically active and dividing, dormant or nonviable at the time of sample collection can be captured during the analysis (although propidium monoazide can be used to deplete relic DNA [24]). Knowing that the metabolic activities of certain microbes could be suppressed by antagonists and so may not be actively involved in a given condition (phenotype), capturing such bacteria due to their sheer presence may give a false positive association. This is supported by studies that have reported that taxonomic abundance does not correspond to transcriptional activities [25,26,27]. The library construction, assembly, and reference databases of metagenomics for annotation employ biases that are difficult to understand [21]. Recently, the challenge of mapping reads back to their source transcripts when working with microbes that lack reference genomes have been minimized by deep whole metagenome sequencing [28]. Yet, as the field of metagenomics advances and more microbes are discovered, the functional annotation steps would continue to develop to meet these needs. Meanwhile, knowing that less than 2% of the genome codes for proteins [29], functional roles predicted from metagenomics data remain inconclusive. Some of the challenges associated with metagenomics studies are however resolved by another technique known as metatranscriptomics.

### 2.2. Metatranscriptomics

Since live gut microbes respond to stress caused by diet, disease, drugs and other external factors, analyzing their RNA would give a clue to their metabolic activities in response to the stress and how such responses influence the host’s phenotype (health or disease condition). Metatranscriptomics therefore exploits RNA sequences to reveal the presence and quantity of microbial RNA in a biological sample at a given time. However, since mRNA make up less than 5% of total RNA of the cell [30], identifying and quantifying gut microbial mRNA will give leads to which genes and pathways are active in the gut ecosystem and play critical roles in health and disease. Recently, De Fillipis et al. [31] found that *Faecalibacterium* species and strains in humans may vary depending on the age, lifestyle and geography of the subjects being studied and that each *Faecalibacterium* clade have distinct functions. Similarly, Ruminococcus gnavus has been found to produce an inflammatory glucorhamnan which can induce dendritic cells to secret TNFα in patients with Crohn’s disease [32]. However, the same bacterium specie has also been reported to have the ability to fortify gut barrier functions by modulating mucin production in the gut [33] which may eventually prevent gut mucosal inflammation [34]. The latter observation is possible because the bacterium may display different activities based on the prevailing conditions (phenotypic switching) [35,36]. For this reason, analyzing the mRNA produced by a microbe at a given time would be a better way to predict the metabolic activities and potential function(s) of the organism rather than making predictions based on its mere presence. Using metatranscriptomics, Granata et al [25] showed that carbohydrate, amino acid, and nucleotide metabolism are severely impaired in the duodenal microbiota of obese patients when compared to non-obese patients. Though the analytical technique is biased towards organisms with higher transcriptional rates, assigning mRNA reads to taxa could unveil critical functions contributed by keystone taxa. There are however several challenges in metatranscriptomics analysis. For instance, the absence of polyA signals makes it challenging to isolate bacteria mRNA and this could result in contamination with rRNA [37]. Currently, there are no standard methods for RNA storage and preparation and this has impact on taxa recovery. Also, though metatranscriptomic data can present researchers with unique insight, there is high variability within the transcriptome of individuals than their metagenomes [38]. Earlier studies have shown that some microbial mRNAs may have weak ribosome binding sites and are therefore poorly translated while only those with strong ribosome binding sites are frequently translated [39]. This implies that not all the bulk (total) microbial mRNA detected by metatranscriptomics may actually be involved in a given host phenotype. Meanwhile, since microbial proteins may have direct effects on other microbes and influence host physiology [40,41], identifying and quantifying gut microbial proteins (metaproteomics) and metabolites (metabolomics) would give a better picture of the role of gut microbes in health and disease. 

### 2.3. Metaproteomics

Metaproteomics employs high-resolution mass spectrometry to identify and measure the levels of expressed proteins [42]. The information obtained is processed by pipelines that eventually match the peptides with metagenomic databases in other to figure out the most probable microbes that might have expressed the proteins. Several studies have used this analytical method to identify gut microbial proteins that directly affect the host or cause dysbiosis. For instance, using metaproteomics Zhang et al. [43] showed that inflammation and oxidative stress associated with inflammatory bowel disease (IBD) induces significant amounts of *Caudovirales* phage proteins which are linked to gut microbial dysbiosis. Similarly, Long et al. [44] reported that oxidative stress associated with colorectal cancer induces excessive bacterioferritin production in *Parabacteroides* which enables them to outgrow other bacteria in the gut resulting in dysbiosis. Additionally, consumption of refined grains was shown to promote the colonization of colonic mucus-degrading bacteria which secrete β-galactosidases and endo-alpha-*N*-acetylgalactosaminidase to degrade mucin thereby impairing gut barrier functions. These studies are few examples of how metaproteomics enables a clear identification of how disease conditions (or their triggers) result in gut microbial dysbiosis and which microbial proteins may directly affect the host. Several platforms such as MetaLab [45], MetaQuantome [46], Galaxy-P [47], and MetaProteomeAnalyzer [48] are available for processing metaproteomic data. The ability of metaproteomics to identify and quantify proteins from microbial and host sources makes it a powerful approach in studying microbial–host interactions [43,49]. However, metaproteomics comes with its own challenges. Apart for variations that exist among microbial proteins obtained from different individuals, microbial proteins could be contaminated with host and undigested food proteins [50] making it difficult to identify the origin of the detected protein. Also, since there is no standardized method for sample preparation for metatproteomic analysis, similar studies may yield varying results [51,52]. Furthermore, not all the detected microbial proteins may be present in the protein database being use for the identification [53] and this can limit the number of peptides identified in a given sample. Meanwhile, there are still many microbial proteins that have not been characterized and have no known functions [54,55] and this can make it difficult to make sense of such data during metaproteomic analysis.

Over the years, several bioinformatic pipelines that use the Lowest Common Ancestor algorithm have been developed to map microbial peptides to taxonomic ranks in other to trace the sources of the peptides identified by metaproteomics [42]. Meanwhile, these pipelines only make inferences based on available databases [51]. Recently studies have shown the tendency for different cells to fuse and exchange cellular materials to yield hybrid cells that express that belong to both parent cells [54] and also considering that different bacteria may produce a given protein [56,57], inferring the source of a protein form a database may not give a good picture of the populations of the various microbes that actually produced the protein. Many studies therefore combine metagenomics and metaproteomic data to enhance the assignment of proteins to taxons in a more realistic way [58]. Meanwhile, since other microbial metabolites apart from proteins influence host health and disease, it is imperative to analyze such compounds for a better understanding of microbial–host interactions.

### 2.4. Metabolomics

Metabolomics involves the study of metabolites in a biological sample at a given time. A targeted or untargeted approach could be used. In targeted metabolomics, metabolites that are involved in specific pathways linked with a specific disease condition are quantified [59]. The untargeted approach however tends to measure as many metabolites as possible from samples without any bias [60]. It is a powerful technique that can unveil the metabolic pattern of the gut microbiota under a given condition [61] and also discriminate between metabolites that may be associated with different conditions [59]. Metabolomics has been applied to detect biomarkers that can distinguish early stage colorectal cancer (CRC) patients from advanced stage CRC patients based on urine metabolites [62]. Similarly, untargeted metabolomics has revealed that lactosylceramide is a key metabolite that can distinguish children with Crohn’s disease from those with ulcerative colitis [63]. Analytical techniques such as liquid chromatography, gas chromatography, mass spectrometry (MS), MS/MS, ultraviolet/visible spectroscopy and nuclear magnetic resonance spectroscopy that are employed in metabolomics studies have already been discussed in our earlier review [64]. Several studies have shown that patient and sample preparation methods can have significant impacts on the gut microbiota [65,66,67] and their metabolites being analyzed [68] and this can cause disparity in the results obtained in similar studies. It is however noteworthy that metabolomics data alone do not give any information about which specific bacteria produced a given metabolite. Also, knowing that different microbes such as *Ruminococcaceae* and *Lachnospiraceae* can produce the same metabolites (eg. short chain fatty acids) [69], it is challenging to accurately predict which specific bacteria produced certain metabolites even when metabolomics data is merged with other “omic” data. Since no single “omic” strategy is enough to present a detailed mechanism to describe gut microbial–host interaction in health and disease, combining several “omic” techniques (multi-omics) is a powerful strategy for making valid conclusions.

## 3. Integrative Multi-Omics for Host–Microbiome Interaction Studies

For deeper information regarding the microbial phylogeny, metabolic potentials, metabolic pathways as well as their actual proteins and metabolites involved in a given host phenotype, a combination of omic techniques have been used in several studies. The commonest integrative multi-omic approaches that have been used over the years for microbiome studies are combinations of metagenomics and metatranscriptomics, metagenomics and metatranscriptomics and metagenomics and metatranscriptomics. These approaches have been discussed below.

### 3.1. Combination of Metagenomics and Metatranscriptomics

The gut microbial communities consist of few dominant communities and a large number of rare taxa [70,71] which may play critical roles in the overall metabolic fluxes associated with health and disease. Since metagenomics does not provide information about the dynamics of gene expression, some studies directly analyze both DNA (metagenomics) and RNA sequences (metatranscriptomics) from gut microbial communities so as to unveil the functional diversity and ecological partitioning of the gut microbiota. Using this combined approach, Lim et al [72] observed that although patients with cystic fibrosis have different gut viral and bacterial compositions, the metabolic activities of their gut microbes are very similar. Conversely, Turnbaugh et al [73] used the combined metagenomics and metatranscriptomics approach to demonstrate that although monozygotic twins may have similar specie-level phylotypes in their guts, there were remarkable differences in gene content and transcriptional activities of their gut microbes. Results from these studies therefore indicate that variations and similarities in the gut microbiota may not be as important as the roles they play in the gut. This is partly because the activities of certain microbes are significantly altered when they transit from one ecological niche to another [38] and hence a combined metagenomics-metatranscriptomic approach is a powerful tool to infer which microbes are metabolically active and which of them are not in health and disease.

### 3.2. Combination of Metagenomics and Metaproteomics

The ability to identify the members of a microbial community and their metabolic potentials as well as their gene expression (metaproteome data) is important to concurrently explore the gut microbiome at several molecular levels [54]. This is because the activities of microbes can easily be predicted by identifying the functions of the proteins they express. For this reason, a number of studies have combined metagenomics and metaproteomics to identify microbial communities and their characteristic proteins expressed in health and disease. By this approach, Erickson et al. [54] observed that Crohn’s disease (CD) associated gut dysbiosis results in the expression of high amounts of microbial antigenic cell wall proteins that exacerbate immune response. They also demonstrated that CD resulted in a significant reduction in microbes and microbial proteins involved in butyrate production and mucin degradation. In another study, it was observed that dysbiosis in type-2 diabetes resulted in a significant increase in microbial carbohydrate metabolizing enzymes and a decrease in the production of host antimicrobial proteins as well as host proteins that enhance gut barrier functions [74]. The study therefore revealed a complex relationship between gut dysbiosis and host response in type-2 diabetes. Meanwhile, since other microbial compounds other than proteins may also affect host health and disease, other studies have studied host–gut microbe interaction by using a combination of metagenomics and metabolomics.

### 3.3. Combination of Metagenomics and Metabolomics

Microbes communicate with their hosts through molecular interactions and this makes a combined metagenomics–metabolomics approach a powerful means of exploring the impact of gut microbes in health and disease. This approach enables microbial biomarker or microbial metabolite identification which could be used for early detection of disease conditions. The combined approach is particularly effective in revealing the impact of different conditions on the gut microbial community and quantitatively measuring their metabolic response. For instance Gual-Grau et al [75] recently demonstrated that consuming a cafeteria diet for 10 weeks could induced obesity. Using a combined metagenomics–metabolomic approach, they observed that the cafeteria diet caused a decrease in the populations of *Clostridium*, *Dehalobacterium*, *Oscillospira,* and *Anaeroplasma* and resulted in an accumulation of hippuric acid (a biomarker of metabolic disease). In another study, the approach was used to study the impact of host gut ecological changes and microbial metabolome on obesity in two different mice strains [76]. The strategy helped to unveil microbial metabolites that were common and those that were unique to each mouse strain during obesity [76]. Such a study points to the importance of personalized nutrition in modulating the gut microbiota of different people with a given disease condition. In studying the fecal microbiota and metabolome of extremely obese patients and normal weight subjects, Nogacka et al. [77] demonstrated that the two groups could be distinguished by their gut microbial profiles as well as their microbial bilirubin metabolism, catecholamines and levels of short chain fatty acids in their fecal samples. By metabolomics analysis of fecal samples, Nogacka et al showed that dietary interventions that induce weigh loss were strongly linked with the gut–brain- and gut–liver axes. We have previously reviewed the application of metagenomics and metabolomics in health and disease [64]. Although much progress has been made in the use of this approach to understand the role of gut microbes in health and disease, challenges still exist in deconvoluting the complex gut metabolites to confirm whether a given metabolite present in a fecal sample is a host, microbial or host–microbe cometabolite. And if the compound is a microbial metabolite, it is very challenging to identify which specific specie produced it [78].

## 4. Future Perspectives

Despite the advances in the application of multi-omics for studying the interaction of gut microbiota and their host, the biological interpretation of data remains challenging. It is still puzzling to identify which specific bacteria species produce certain specific metabolites which may be associated with health and disease. In fact, adequate information on the source (whether microbial or host) of metabolites and knowledge of the possible pleiotropic effects will be needed in other to make research findings useful in the development of therapeutics strategies for a given disease condition. To accomplish this, advanced analytic frameworks that exhaustively process multiple types of omic data sets is required in this area of study. In future, researchers may need to combine several omic-techniques rather than relying on just one or two techniques so as to make up for the weaknesses of each method. Such an approach would be a crucial step toward achieving precision nutrition or medicine and help in understanding the precise mechanism(s) by which the gut microbiota is in health and disease.
